# Non-active site mutants of HIV-1 protease influence resistance and sensitisation towards protease inhibitors

**DOI:** 10.1186/s12977-020-00520-6

**Published:** 2020-05-19

**Authors:** Tomas Bastys, Vytautas Gapsys, Hauke Walter, Eva Heger, Nadezhda T. Doncheva, Rolf Kaiser, Bert L. de Groot, Olga V. Kalinina

**Affiliations:** 1 Department for Computational Biology and Applied Algorithmics, Max Planck Institute for Informatics, 66123 Saarbrücken, Germany; 2 Saarbrücken Graduate School of Computer Science, University of Saarland, 66123 Saarbrücken, Germany; 3 Computational Biomolecular Dynamics Group, Department of Theoretical and Computational Biophysics, Max Planck Institute for Biophysical Chemistry, 37077 Göttingen, Germany; 4 Medizinisches Labor Stendal, 39576 Stendal, Germany; 5 Institute of Virology, University of Cologne, 50935 Cologne, Germany; 6 Faculty of Health and Medical Sciences, University of Copenhagen, 2200 Copenhagen, Denmark; 7 Helmholtz Institute for Pharmaceutical Research Saarland (HIPS), Helmholtz Centre for Infection Research (HZI), 66123 Saarbrücken, Germany; 8 Faculty of Medicine, Saarland University, 66421 Homburg, Germany

**Keywords:** Alchemical binding free energy change calculation, Distant site mutations, HIV-1 protease inhibitors, Hydrogen bond network perturbation, Resistance-associated mutations

## Abstract

**Background:**

HIV-1 can develop resistance to antiretroviral drugs, mainly through mutations within the target regions of the drugs. In HIV-1 protease, a majority of resistance-associated mutations that develop in response to therapy with protease inhibitors are found in the protease’s active site that serves also as a binding pocket for the protease inhibitors, thus directly impacting the protease-inhibitor interactions. Some resistance-associated mutations, however, are found in more distant regions, and the exact mechanisms how these mutations affect protease-inhibitor interactions are unclear. Furthermore, some of these mutations, e.g. N88S and L76V, do not only induce resistance to the currently administered drugs, but contrarily induce sensitivity towards other drugs. In this study, mutations N88S and L76V, along with three other resistance-associated mutations, M46I, I50L, and I84V, are analysed by means of molecular dynamics simulations to investigate their role in complexes of the protease with different inhibitors and in different background sequence contexts.

**Results:**

Using these simulations for alchemical calculations to estimate the effects of mutations M46I, I50L, I84V, N88S, and L76V on binding free energies shows they are in general in line with the mutations’ effect on $IC_{50}$ values. For the primary mutation L76V, however, the presence of a background mutation M46I in our analysis influences whether the unfavourable effect of L76V on inhibitor binding is sufficient to outweigh the accompanying reduction in catalytic activity of the protease. Finally, we show that L76V and N88S changes the hydrogen bond stability of these residues with residues D30/K45 and D30/T31/T74, respectively.

**Conclusions:**

We demonstrate that estimating the effect of both binding pocket and distant mutations on inhibitor binding free energy using alchemical calculations can reproduce their effect on the experimentally measured $IC_{50}$ values. We show that distant site mutations L76V and N88S affect the hydrogen bond network in the protease’s active site, which offers an explanation for the indirect effect of these mutations on inhibitor binding. This work thus provides valuable insights on interplay between primary and background mutations and mechanisms how they affect inhibitor binding.

## Background

With around 36.7 million people already infected and 1.8 million being newly infected per year, the human immunodeficiency virus type 1 (HIV-1) (further HIV) remains a global epidemic [1]. Since more than half of the infected individuals receive antiretroviral therapy (ART) [2], acquired immune deficiency syndrome (AIDS)-related deaths have dropped to 1 million per year [1]. For those under treatment, resistance towards drugs is a major cause for the need for switching of the therapy.

**Fig. 1:**
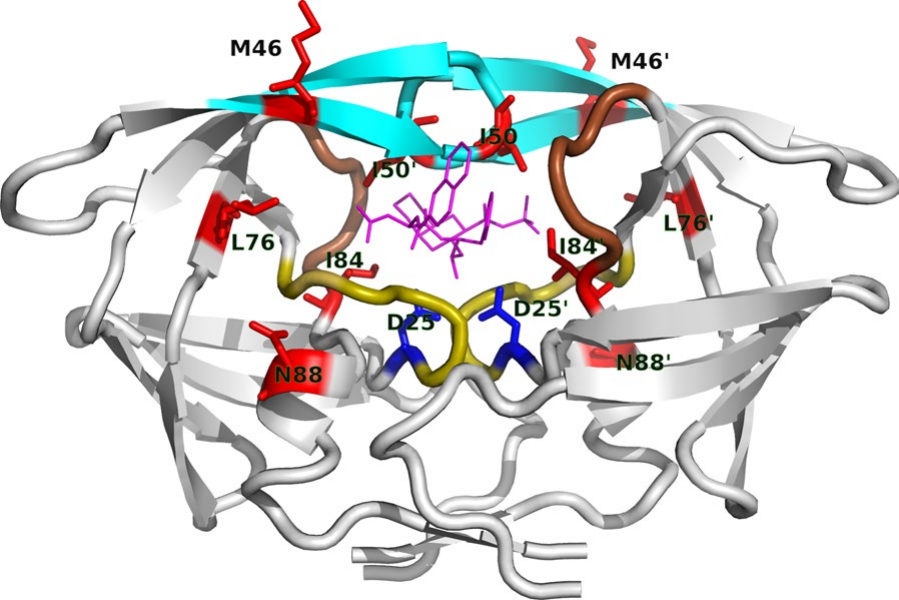
HIV protease structure. Flap region in cyan, 80s loop in brown, active-site proximate loop in olive colours. Mutations analysed in this study (red), catalytic site residue (blue) and bound inhibitor (magenta) are shown in sticks model

HIV protease (Fig. 1), a protein responsible for cleaving HIV polyproteins, is one of the major targets for ART, and protease inhibitors (PIs) are currently recommended as second- or third-line ART treatments [3]. PIs are competitive binders of the protease, occupying the active site of the protein once bound. In line with this, most of the major resistance-associated mutations (RAMs) towards PIs appear in the different structural elements composing the active site pocket, such as in the active site loop (residues D30, V32, and L33), the so-called 80s loop (residues V82 and I84) which together form the sides of the pocket, and the flap region of the protease (residues M46, I47, G48, I50, and I54, Fig. 1). Yet several RAMs are also found in distant to the binding pocket sites, e.g. in the amino acids N88 and L90 in the protease’s $\alpha$-helix or L76 in the protein’s hydrophobic core. The effect of these mutations on inhibitor binding is likely to be not through direct interactions with PIs.

Some mutations exhibit opposite effects on binding of some PIs, e.g. L76V is associated with resistance towards Amprenavir (APV), Indinavir (IDV), Darunavir (DRV), and Lopinavir (LPV), but increases sensitivity towards Atazanavir (ATV) and Saquinavir (SQV) [4, 5]. Similarly, N88S is a RAM towards IDV and Nelfinavir (NFV), but increases susceptibility towards APV [6–8] or its prodrug Fosamprenavir (FPV) [9].

Numerous studies have addressed the molecular effects of RAMs. Some studies analyse the effects of selected major RAMs on binding of different inhibitors [10–17] and others the effect of different RAMs on binding of the same inhibitor [18–27]. Most of the studies are however focused on a single mutation-inhibitor combination, particularly for major RAMs outside of the binding pocket, and thus offer only a limited perspective on molecular mechanisms of the protease resistance. To the best of our knowledge, the mechanism of action of a mutation on binding of different inhibitors has not been investigated in the aforementioned cases, where the same mutation is known to cause resistance to certain inhibitors, while making the protein sensitive towards other inhibitors [4–9]. In clinical practice of HIV treatment, cases like this potentially provide an opportunity for combined treatment: a combination of PIs that are associated with opposite effects of a particular mutation put the virus in a situation where either mutation variant in this position will render the protease susceptible to one of the drugs [5, 6, 28]. Understanding the underlying molecular phenomena could potentially provide important insights into HIV inhibitor resistance, as well as a possibility to transfer this knowledge to treatment of other viruses, and for inhibitor design.

In an experimental setting, resistance of mutant proteins towards PIs, such as in the studies above, is typically measured in terms of $IC_{50}$ (concentration required to inhibit viral activity by 50%). Thus, the ratio between $IC_{50}$ in mutant and the same measurement for the wildtype protease (typically with the consensus sequence from the strain HXB2), also called resistance factor (RF), is a useful descriptor for resistance of different mutated proteins. RF is directly related to the free energy of inhibitor binding, $\Delta G$, and the protease enzymatic activity, $K_m$ [29].

We have previously shown that the effect of mutations in the HIV protease on inhibitor binding, $\Delta \Delta G$, can be accurately predicted in silico using alchemical methods based on molecular dynamics (MD) simulations [17]. Additionally, analysis of the underlying trajectories from the MD simulations can also reveal the mechanisms underlying the effects of mutations on protein-inhibitor interactions, protein structural changes, as well as on their altered dynamics.

In this study, we apply alchemical calculations based on MD simulations to estimate the effect of RAMs M46I, I50L, I84V, N88S, and L76V, both in the binding pocket and outside of it, on the binding of PIs APV, IDV, LPV, and SQV: in total 19 different PI-mutation combinations. We demonstrate that we can faithfully reproduce the previously reported effects of the first four mutations on the resistance, including previously investigated sensitising and desensitising effects of the mutation N88S on binding of APV and IDV [6, 8]. We show that changes of the hydrogen bonding network of the protease that involve D30, located in the active site pocket, can explain these effects.

The data on M46I, I50L, I84V, and N88S were acquired from the HIVdb database [30]. Measurements can be paired such that the protease sequences are identical with the exception of the mutated site. Moreover, for all of these mutations wildtype and mutant protein background sequences were identical across the different inhibitors, with the exception of N88S, where complexes with FPV had a background mutation L77I and complexes with IDV had a R57G background mutation present. Both of R57G and L77I mutations are found next to each other on two parallel $\beta$-strands at a distant site of the protease and close to major RAM sites I54 and L76, respectively. However, unlike in case of the latter residues, the side chains of residues 77 and 57 are pointing away from the protease binding pocket. Nevertheless, the mutation R57G has been suggested to be a protease-inactivating mutation [31]. L77I on the other hand, has been reported to be a compensatory mutation for I84V, restoring protein stability [32]. M46I, I84V, and N88S are all considered to be major RAMs against the corresponding inhibitors as reported in the HIVdb. In addition to that N88S has been previously reported to increase susceptibility against APV/FPV [6–9]. I50L on the other hand has been reported to induce resistance against ATV while increasing sensitivity against remaining PIs [33–36].

For L76V, located in the protease’s hydrophobic core, we investigate its effect on binding of ATV, IDV, LPV, and SQV in different clinically relevant sequence contexts. In the phenotypic assays we consistently observe a resensitising effect of this mutation towards ATV and SQV as well as its resistance-associated effect for IDV and LPV, which was reported previously [4, 5], and we generally reproduce these effects computationally. We suggest a mechanistic explanation of this effect, demonstrating that these mutations affect the arrangement of residues around the binding pocket of the protease, which, in turn, similarly to N88S, affects the hydrogen bonding network of D30, directly influencing inhibitor binding.

## Results and discussion

### Estimation of resistance factors from the change in the inhibitor binding free energy

We aimed to assess whether we can reproduce the ratios of experimentally measured resistance factors ($RF_R = RF_{mutant}/RF_{wildtype}$) between the wildtype and mutant proteins by estimating the change in free energy of inhibitor binding upon mutations in the protease using MD simulation with alchemical methods [37]. For this purpose we selected a dataset of 20 complexes from HIVdb database [30] for which resistance factors towards inhibitors FPV, IDV, LPV, and SQV had been measured experimentally (Table 1 and Additional file 1: Table S1). These complexes could be paired amongst each other such that the RF has been measured for the same inhibitor and the same protease strain with and without the mutation, namely: IDV and FPV with mutation M46I; IDV and FPV with mutation I50L; FPV, IDV, LPV, and SQV with mutation I84V; FPV and IDV with mutation N88S. For all of these pairs, protease with a RAM had a higher RF than the wildtype, except I50L reducing resistance towards FPV and IDV and N88S reducing resistance to FPV (Table 1).

**Table 1:** RF values for different mutant and corresponding wildtype sequences from HIVdb [30]

Mutation		Background polymorphisms	RF			
			IDV	SQV	LPV	FPV
M46I						
Wildtype [33]		V3I, S37N, A71V	0.6, 1.0, 1.2	–	–	0.3, 0.6, 0.7
Mutant [83]			4.4	–	–	2.2
I50L						
Wildtype [33]		V3I, S37N, A71V	0.6, 1.0, 1.2	–	–	0.3, 0.6, 0.7
Mutant [33]			0.1, 0.3, 0.3	–	–	0.2, 0.2, 0.3
I84V						
Wildtype [84]		V3I, L10F, S37N	1.5	1.3	1.6	1.8
Mutant [84]			2.1, 3.2	2.7, 3.7	6.2, 7.7	4.6, 8.4
N88S						
Wildtype [6]		V3I, S37N, L63P, R57G/L77I^a^	1.1	–	–	1.0
Mutant [6]			2.6	–	–	0.1

First column indicates the mutation analysed, while the second column indicates background polymorphisms that are present in both wildtype and mutant sequences compared to the reference HIV sequence HXB2. Multiple RF measurements for the same protein are separated by comma. M46I wildtype and mutant measurements reported from different studies, but performed using the same susceptibility test method (Phenosense, Monogram, San Francisco, USA)

^a^ Indicates that the R57G background mutation was found in sequences where RF for IDV was measured and L77I in sequences where RF for FPV was measured

We first estimated the effect of the mutation on the free energy of inhibitor binding, $\Delta \Delta G$. For this purpose, we performed MD simulations for the protease mutants and wildtype in inhibitor-bound and unbound state, where in the bound state the simulations were performed in both alternative protonation states of the catalytic residues of the active site, D25 and D25$^\prime$, to account for asymmetry of this complex. This allows us to identify which protonation state is more likely for both wildtype and mutant complexes, as well as to increase the accuracy of the $\Delta \Delta G$ estimation, as we reported previously [17]. The resulting $\Delta \Delta G$ calculations (Table 2 and Additional file 1: Table S2) overall indicated a good agreement in discriminating resistant and sensitising effects of mutations on the protein–ligand binding, including the opposite effects of N88S towards IDV and APV. An exception to that is M46I, where the mutation had a modest effect on $\Delta G$ which was within the estimated error range. The mutation of this flap residue, whose side-chain points away from the protease binding pocket, has been associated with resistance towards different PIs, but it typically appears in combination with other RAMs and has been suggested to compensate the decreased catalytic activity of mutant proteases [38–43].

**Table 2:** Change of the binding free energy ($\Delta \Delta G$) of inhibitors upon mutation

Inhibitor	Mutation	$\Delta \Delta G$	$RF^{exp}_R$	$RF^{calc}_R$
APV	M46I	$-0.35 \pm 0.4$	3.14–7.33^a^	13.13$^{77.54}_{0.3}$
IDV	M46I	$-0.34 \pm 0.72$	3.67–7.33	12.69$^{75.84}_{0.27}$
APV	I50L	$-0.64 \pm 0.41$	0.29–1^a^	0.74$^{3.87}_{0.03}$
IDV	I50L	$-0.87 \pm 0.51$	0.08–0.5	0.62$^{3.17}_{0.03}$
APV	I84V	$2.06 \pm 0.47$	2.56–4.67^a^	33.75$^{169.66}_{1.25}$
IDV	I84V	$1.13 \pm 0.57$	1.4–2.13	7.23$^{42.3}_{0.11}$
LPV	I84V	$1.25 \pm 0.39$	3.88–4.81	6.09$^{33.62}_{0.17}$
SQV	I84V	$0.56 \pm 0.41$	2.08–2.85	1.33$^{6.73}_{0.05}$
APV	N88S	$-0.97 \pm 0.7$	0.1^a^	0.3$^{2.14}_{0.001}$
IDV	N88S	$1.41 \pm 0.95$	2.27	41.12$^{281.92}_{0.11}$

All values in kcal/mol, and ± shows bootstrap error estimate. $RF^{exp}_R$ indicates value ranges calculated using $RF_R = RF_{mutant}/RF_{wildtype}$ from previously reported experimental RF measurements, where "^a^" stands for measurements for FPV, the prodrug of APV. $RF^{calc}_R$ indicates average calculated $RF_R$ value from the distribution described in Eq. 4 with subscript and superscript corresponding to lower and upper bound of 95% credible interval, respectively

The possibility of mutations having an effect on the catalytic activity of the enzyme, $K_m$, precludes direct comparison of the $\Delta \Delta G$ estimates of mutation effects on inhibitor binding and the $RF_R$ corresponding to that mutation. In previous studies of resistance mutations of another enzyme of HIV, reverse transcriptase, $\Delta \Delta G$ was considered to approximate changes in $IC_{50}$ [44–46]. This is at odds with the fact that the majority of the mutations for which this approximation was used, such as L100I, V106A, and Y188L, although not located directly in the active site, have been previously reported to affect the catalytic potential of the enzyme [47–51]. Although some studies show correlation between predicted relative drug binding free energy upon HIV protease mutation and the one approximated by $IC_{50}$ measurements [52], RAMs affecting the catalytic activity of protease have also been reported [39, 40, 53, 54]. In the present study, similarly to the aforementioned computational studies, only the experimentally measured $IC_{50}$ values for mutations are available for the enzyme and different inhibitors. To account for the changes in the binding free energy and $K_m$, we developed a Bayesian method which combines multiple experimental $RF_R$ measurements and $\Delta \Delta G$ estimates to calculate $RF_R$ (see “Estimation of resistance factor change from free energy of inhibitor binding change”).

**Fig. 2:**
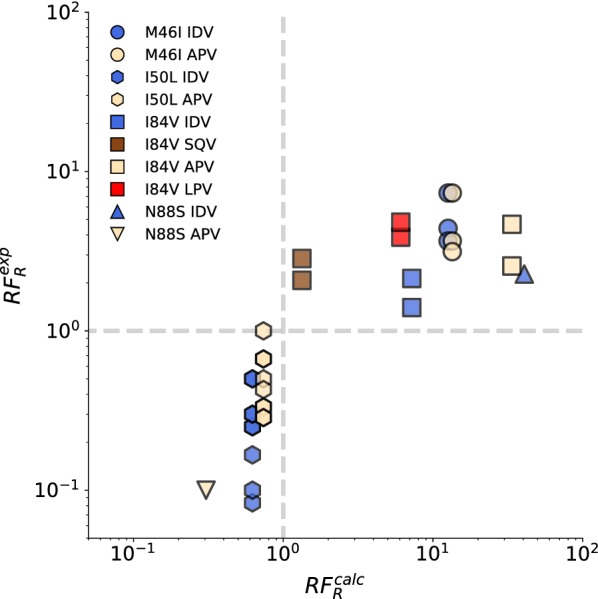
Predicted and experimental RF measurements. Each symbol corresponds to a unique sequence background and colour corresponds to inhibitor. In case of APV, $RF^{exp}_R$ measurements are for its prodrug FPV

We then compared the estimated $RF_R$ values to their experimental measurements (Table 2, Fig. 2). The increase of resistance towards inhibitors ($RF_R>1$) was correctly predicted for M46I, I84V, and N88S (with IDV) mutations, as was the sensitising effect ($RF_R<1$) of I50L towards FPV and IDV as well as N88S towards APV. The experimental $RF_R$ values were within the corresponding calculated distributions based on the $\Delta \Delta G$ estimates (Additional file 1: Figure S1).

While the structural effect of N88S mutation has been previously analysed for NFV, to the best of our knowledge, the opposite effects of this mutation on susceptibility towards APV and IDV have not been previously addressed. It has been suggested that substitution of asparagine with serine creates a hydrogen bond with the residue D30, which in turn affects the interaction between D30 and the inhibitor NFV [55]. A similar observation with regard to the N88S effect on the interaction with D30 has been previously made for L10F/N88S mutant with NFV as well as the unliganded N88S protease [56, 57]. Another mutation at this site that occurs in patients treated with NFV, N88D, has been reported to co-occur with mutation D30N, which coincides with losing water molecules that mediate this site’s interactions with residues T31 and T74 [58]. Seeking to verify whether these effects extend to complexes of N88S with APV and IDV, we performed hydrogen bond network analysis of mutant and wildtype complexes, where we measured the average number of hydrogen bonds over the course of the simulations. Indeed, similar effects were confirmed: S88 formed a hydrogen bond with D30 more frequently than N88, whereas N88 formed a hydrogen bond more frequenly to T31 and T74 compared to S88 (Additional file 1: Table S3).

### Estimating resistance factor for clinical samples with the L76V mutation

Among patients in Germany undergoing HIV treatment, individuals who underwent multiple therapy failures against different PI-regimens were identified. Among the group of patients we described in the manuscript of Wiesman et al. [5], there were viral variants which displayed different resistance-determining mutations to ATV, SQV, IDV, and LPV. The extraordinary observation was that a specific amino acid change L76V increased resistance to LPV and IDV, while at the same time giving a clinically relevant re-sensitisation to SQV and ATV. Those variants were observed in the diagnostic procedure, sequenced, and subsequently tested in a phenotypic assay. We analysed variants which showed some of the highest changes in RF upon mutation to evaluate whether we can computationally estimate RF values as measured in the phenotypic assay (Table 3).

**Table 3:** Protease RF values for L76V mutation

Genotype		RF			
		ATV	SQV	IDV	LPV
FB15					
L76		63	74	–	–
V76		0.9, 2.3, 4.5	3.6, 4.6, 5.8	–	–
GH9					
L76		90	18.6	–	–
V76		1.2, 1.9, 2.4, 3.2, 3.6	1.2, 1.3, 1.5	–	–
RU1					
V76		2.7	–	–	157
L76		8.4, 9, 10	–	–	27, 46, 47
iZ2					
V76		4.1	–	59	71
L76		7.8, 12, 30	–	2.2, 7.9, 10	5.5, 11, 12

Multiple RF measurements for the same protein separated by comma. For each genotype the first row represents the wildtype position as in the original sample and the second row represents the mutation introduced at position 76

Just as for mutations M46I, I50L, I84V, and N88S, for the mutation L76V multiple RF measurements for different inhibitors in the same sequence context were available, thus enabling us to computationally predict the $RF_R$ values. The sequences of the protease complexes analysed had a large number of background mutations accumulated compared to the reference sequence HXB2, making it difficult to find complexes in the Protein Data Bank (PDB) with sequences matching the studied genotypes. Thus in the protein modelling stage between 11 and 19 mutations had to be introduced to create protein models with sequences corresponding to those for which $RF_R$ was measured (see “System preparation”). Including the target mutation L76V as well meant that up to 20% of protease residues had to be modelled in silico.

**Table 4:** Change of the binding free energy ($\Delta \Delta G$) of inhibitors upon mutation L76V

Inhibitor	Genotype	$\Delta \Delta G$	$RF^{exp}_R$	$RF^{calc}_R$
ATV	FB15	$0.78 \pm 0.64$	0.01–0.07	0.23$^{1.41}_{0.004}$
SQV	FB15	$0.69 \pm 0.62$	0.04–0.08	0.11$^{0.71}_{0.001}$
ATV	GH9	$-0.29 \pm 0.49$	0.01–0.04	0.23$^{1.12}_{0.01}$
SQV	GH9	$-0.58 \pm 0.47$	0.06–0.08	0.03$^{0.17}_{0.001}$
ATV	RU1	$0.74 \pm 0.73$	0.27–0.32	3.52$^{22.7}_{0.04}$
LPV	RU1	$1.52 \pm 0.6$	3–5	3.94$^{25.08}_{0.05}$
ATV	iZ2	$1.11 \pm 0.65$	0.14–0.53	35.61$^{243.1}_{0.17}$
IDV	iZ2	$1.76 \pm 0.66$	5.9–26.82	88.25$^{649.19}_{0.06}$
LPV	iZ2	$0.99 \pm 0.88$	5.91–12.9	10.95$^{76.68}_{0.01}$

All values in kcal/mol, and ± shows bootstrap error estimate. $RF^{exp}_R$ indicates value ranges calculated using $RF_R = RF_{mutant}/RF_{wildtype}$ from experimental RF measurements, $RF^{calc}_R$ indicates average calculated $RF_R$ value from the distribution described in Eq. 4 with subscript and superscript corresponding to lower and upper bound of 95% credible interval, respectively

First, we estimated the effect of the mutation L76V on inhibitor binding in terms of the change of the binding free energy $\Delta \Delta G$ (Table 4 and Additional file 1: Table S4). The increase of the binding free energy, corresponding to the decrease in inhibitor affinity, was predicted for all complexes where mutations were observed to increase the protease RF (RU1 with LPV, iZ2 with IDV and LPV). The decrease of RF, on the other hand, did not always correspond to a negative value of $\Delta \Delta G$: L76V was predicted to increase the affinity of inhibitor binding for inhibitors ATV and SQV only for the genotype GH9, but not for the genotype FB15, nor for inhibitor ATV in the context of the genotypes RU1 or iZ2. The genotypes FB15 and iZ2 lack the background mutation M46I (the former being wildtype at that position and the latter having mutation M46L), which has been suggested to co-occur with L76V to compensate for its compromising effect on the replication capacity of HIV [41, 42]. This suggests that in this case the dominant effect of the mutation L76V might be exerted through decreasing the protease’s catalytic activity $K_m$. However for RU1, which has the M46I mutation, we cannot explain the positive $\Delta \Delta G$ value for complexes with ATV using this argument.

**Fig. 3:**
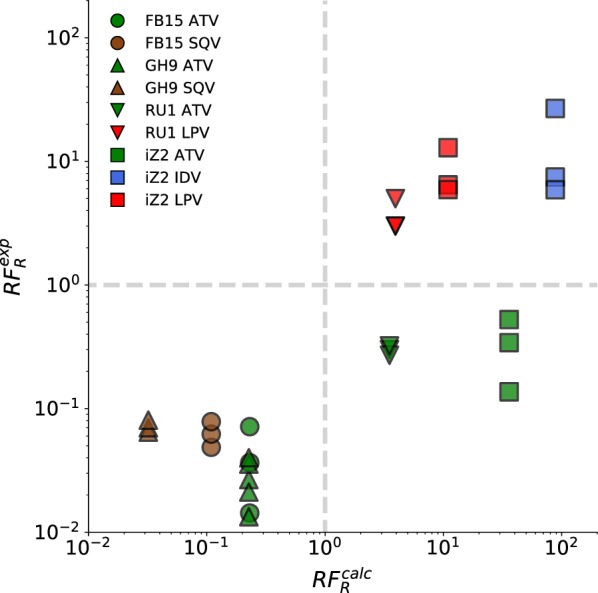
Predicted and experimental RF measurements. Each symbol corresponds to a unique sequence background and colours correspond to inhibitor

The $\Delta \Delta G$ estimates were used to calculate $RF_R$ in the same fashion as for mutations M46I, I50L, I84V, and N88S (Table 4, Fig. 3). For most of the complexes we correctly predicted whether the mutation made the protein more resistant or more sensitive towards the inhibitor. This included the prediction of sensitising effect of mutation in the genotype FB15 for both ATV and SQV for which the inhibitor affinity increased based on the $\Delta \Delta G$ estimates. Sensitisation towards ATV was, on the other hand, not observed for genotypes RU1 and iZ2. The experimental $RF_R$ values were however within the corresponding calculated distributions based on the $\Delta \Delta G$ estimates (Additional file 1: Figure S2). Overall, just like in the case for M46I, I50L, M84I, and N88S mutations, $RF_R$ estimates converged roughly after half of simulation time (Additional file 1: Figure S3).

### Effect of L76V on molecular interactions

**Fig. 4:**
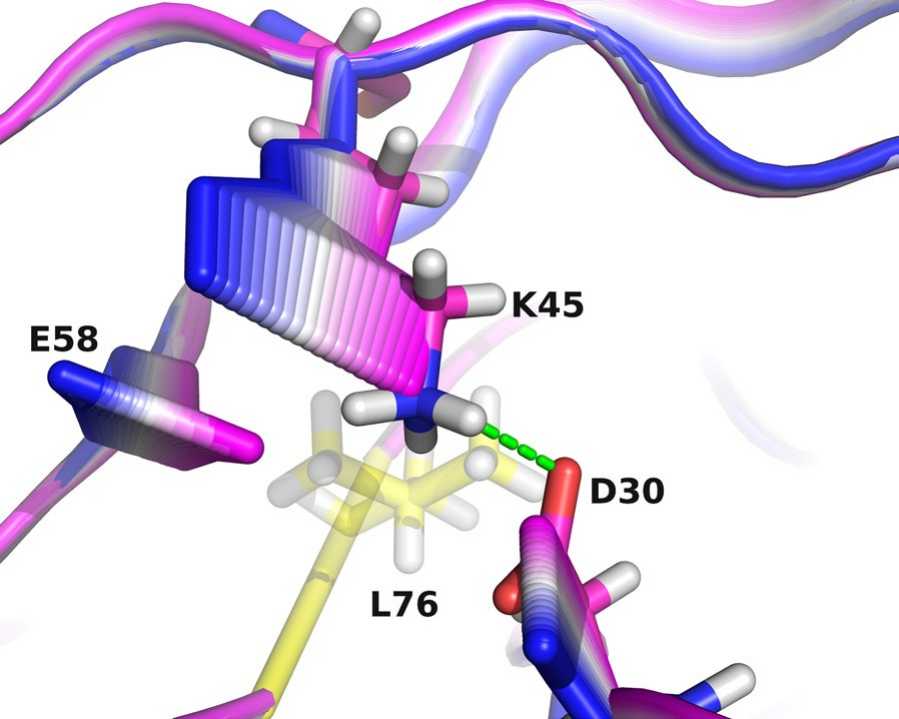
Interpolation between the extremes of the FMA models for the protease (genotype RU1) in complex with LPV. Blue-to-magenta bands correspond to the interpolation along the mode as represented as cartoon for backbone and as sticks for residues 30, 45, and 58, with blue corresponding to L76 state and magenta to V76 state. Green dashed line represents a hydrogen bond between residues D30 and K45. Mutated residue 76, here semi-transparent in yellow, as well as hydrogen atoms, here in gray, were not part of the FMA models and are here for representational purposes only

Next, we focused on the structural changes in the protease upon the mutation L76V, for which purpose we first analysed the hydrogen bond network of the protein. It was consistently seen across all of the different genotypes that the mutation L76V increases the probability of observing a hydrogen bond between residues D30 and K45 (Table 5). Previous studies found a significant correlation between mutations in these sites [59, 60], potentially indicating the importance of the interaction between these two oppositely charged residues. Seeking whether this was a result of side chain rearrangement, we performed functional mode analysis (FMA) based on partial least-squares (PLS) regression, a supervised machine learning technique which builds models that distinguish behaviour in MD trajectories between the wildtype and mutant protease complex based on their Cartesian atoms coordinates. These models are interpretable in terms of protein conformational changes associated with the mutation. In analysing FMA models, we could see that the mutation L76V caused a tendency of the side chains of residues D30, K45, and Q/E58 to shift towards the binding pocket (Fig. 4 and Additional file 1: Figure S4). This shift is likely the result of fine rearrangement of residues in the region as a consequence of a larger side chain of leucine being replaced by a smaller valine. The effect of L76V on D30–K45 hydrogen bond is thus similar to the effect of the other distant site mutation we analysed in this study, N88S, on hydrogen bonding of D30. Mutations at site N88 have been reported to be correlated to mutations of D30 and K45 [59, 60]. Displacement of Q/E58, on the other hand, is in line with the previously reported co-occurance of mutations L76V and Q58E [61], and both of these mutations were found in the patient sample RU1. L76V has recently been reported to increase the distance between $C_\alpha$ atoms of residues 16 and 62 on the surface of the protease when in complex with DRV [62]. The same effect on the structure was observed in both the resistance-inducing and the sensitising cases in that study, which is in line with the consistent observation of side-chain rearrangement we report here.

**Table 5:** Average number of hydrogen bonds between residues D30 and K45 for protease wildtype and mutant complexes

Drug	Genotype	D30–K45		D30$^\prime$–K45$^\prime$	
		L76	V76	L76	V76
ATV	FB15	$0.068\pm 0.003$	$0.52\pm 0.04$	$0.07 \pm 0.002$	$0.58 \pm 0.02$
SQV	FB15	$0.12 \pm 0.01$	$0.59\pm 0.02$	$0.11 \pm 0.005$	$0.62 \pm 0.004$
ATV	GH9	$0.07 \pm 0.01$	$0.49\pm 0.12$	$0.06 \pm 0.002$	$0.66 \pm 0.12$
SQV	GH9	$0.07 \pm 0.004$	$0.46\pm 0.04$	$0.01 \pm 4\times 10^{-5}$	$0.19 \pm 0.04$
ATV	RU1	$0.05 \pm 0.001$	$0.43\pm 0.05$	$0.04 \pm 1\times 10^{-4}$	$0.61 \pm 0.09$
LPV	RU1	$0.01 \pm 3\times 10^{-5}$	$0.16\pm 0.03$	$0.16 \pm 0.006$	$0.56 \pm 0.12$
ATV	iZ2	$0.16 \pm 0.007$	$0.57\pm 0.07$	$0.08 \pm 0.002$	$0.52 \pm 0.04$
IDV	iZ2	$0.1 \pm 0.01$	$0.39\pm 0.03$	$0.06 \pm 0.002$	$0.51\pm 0.07$
LPV	iZ2	$0.04 \pm 5\times 10^{-4}$	$0.19\pm 0.02$	$0.24 \pm 0.01$	$0.63\pm 0.02$

± Indicates standard error of bond frequency across independent simulations

**Fig. 5:**
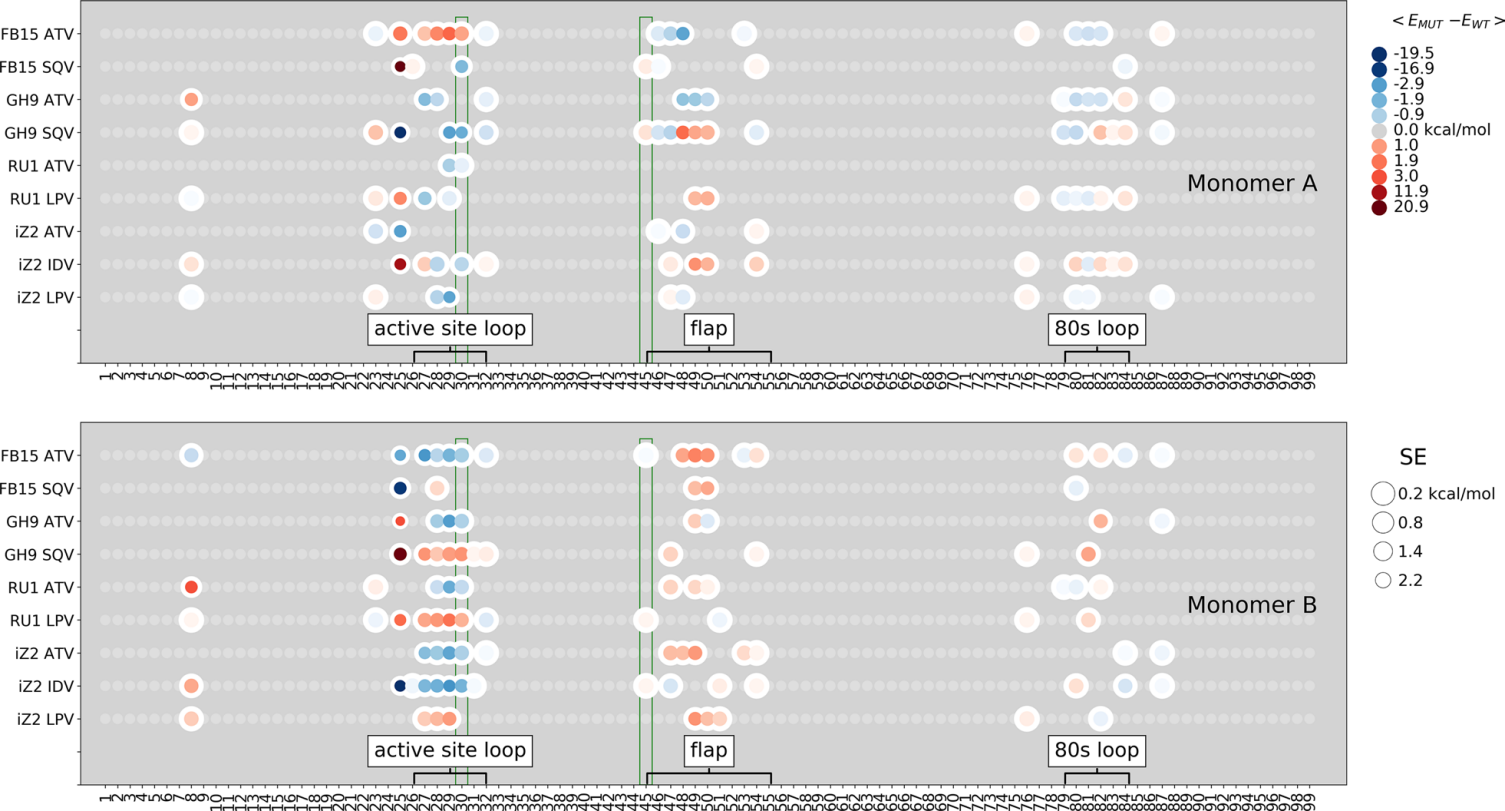
Energy differences of non-bonded interaction between protein and inhibitor in wildtype and mutant complexes. Residues, for which the difference ($E_{MUT}-E_{WT}$) between the wildtype and the mutant complexes is higher than the propagated error (SE) and its absolute value higher than 0.1 kcal/mol, are represented as a colored circle, where the color represents relative interaction energy and the size of the circle relates inversely to the standard error of the estimate. Residues’ 30 and 45 interactions are highlighted in green box

We calculated direct protein-inhibitor interaction energies to see whether the L76V mutation impacted direct interactions of D30, K45, or other residues with the inhibitors (Fig. 5 and Additional file 1: Figure S5). Indeed, changes in the interaction of D30/D30$^\prime$ with the inhibitors are in general in line with changes of $RF_R$: negative, or favourable, interaction energy values correspond to $RF_R<1$, and positive, or unfavourable, interaction energies correspond to $RF_R>1$. Exceptions to that are the proteases of the iZ2 genotype in complex with IDV, where a favourable effect on the direct interaction energy of D30/D30$^\prime$ with the inhibitor is observed, and in complex with LPV, where no notable effect on this interaction is seen. The effect of L76V on interaction energies between the inhibitors and K45/K45$^\prime$ was, on the other hand, modest. A number of other residues’ direct interactions with inhibitor were affected. These residues are widely distributed across most of the active site pocket, including the active site loop (including the D30), flap (including the K45), and 80s loop regions. This is in line with our observations from a previous study of the effect of mutations I50V, G48V, and L90M on protein-inhibitor interactions, where interactions of residues in these regions were also affected by the mutations [17]. Interestingly, measurable differences were observed for interactions of the residue at position 76 with the inhibitor for complexes of genotype FB15 with LPV and ATV and genotype iZ2 with IDV. But given that side chain of residue 76 has minimal exposure to the binding pocket, those differences are negligible.

Overall, these results indicate a pathway for how the mutation L76V impacts the inhibitor binding through altering the interactions of other residues with the inhibitor without actual mutations at these sites. A similar observation has been previously made for another pair of an active site loop and distant site mutations, namely for mutation L90M that alters the interactions of the residue at this position with D25, which in turn affects the interactions of D25 with the inhibitor as well as with other residues in the binding pocket [17, 63, 64]. Our observations on energetic and structural consequences of the mutation L76V are also in line with its previously reported effects on the ligand binding affinity for the inhibitor DRV through both changes in protein-inhibitor interactions and changes in the inter-residue distances in the binding pocket [26].

Recently, a study reported experimentally resolved structures of the wildtype and the L76V mutant of the HIV protease in complex with inhibitors DRV, LPV, Tipranavir (TPV), as well as with two experimental compounds, GRL-0519 and GRL-5010 [65]. It has been observed that mutation does not change the backbone structure of the protease, however residue 76 loses contacts with D30 and T74, and, for structures with LPV, there is a slight shift of K45 towards the binding pocket in the mutant structure. Overall, similar interactions were reported between wildtype and mutant proteins with different inhibitors, with the exception of GRL-5010, which interacted with D30$^\prime$ in an altered way. These results thus partially support the observations made in our study on the effects of the L76V mutation on the structure and interactions within the HIV protease.

## Conclusions

In this work, we analysed a set of mutations in the HIV protease associated with resistance towards PIs in complex with different clinically used inhibitors. First, we analysed four mutations with resistance factors extracted from the literature, where resistance factor measurements for the same sequence and the same inhibitors were available from multiple experiments. We showed that the effect of the mutation on the resistance factor, both increasing resistance and sensitising, was successfully reproduced using alchemical free energy calculations of affinity of inhibitor binding. Second, we modelled complexes for sequences based on our own clinical samples containing the mutation L76V with four PIs. Even though the sequences in question had a large number of background mutations, we could in most cases reproduce the resistant and sensitising effects of L76V. These calculations gave us insight into whether change in resistance is predominantly the result of change in inhibitor binding affinity or a change in the catalytic activity of the protease, for example for sequences which lacked the compensatory mutation M46I. Further analysis of L76V in different sequence contexts revealed that the effect of this mutation on direct protein residue-inhibitor interactions, including that of D30, is generally line with the changes in the resistance. Potentially causal to the observed changes is the favourable effect of the mutation on the hydrogen bond stability between residues D30 and K45 of the binding pocket. Analysis of another distant site mutation, N88S, also revealed changes in hydrogen bonding of the mutated residue with D30 as well as with T31 and T74, suggesting changes in hydrogen bonding network of the protease as a major pathway for how mutations outside of the binding pocket affect inhibitor binding.

## Methods

### Computational studies

#### System preparation

Crystal structures of protease-inhibitor complexes were obtained from the PDB [66] (IDs 1HPV (APV), 1HXB (SQV), 1K6C (IDV), 1MUI (LPV), 2BPX (IDV), 3EKV (APV), 3EL1 (ATV), 3PWR (SQV)). Modeller [67] version 9.12 was used to introduce mutations targeted in this study as well as the background mutations. The following background mutations were introduced in the studied protein from HIVdb dataset: K7Q, R14K, R57G, T82V, and V84I in 1K6C; K7Q, R14K, K41R, and V77I in 3EKV; L10F and S37N in 1MUI; L10F and S37N in 1HPV (84V); S37N and A71V in 1HPV (46V and 50L); L10F and S37N in 2BPX (84V); S37N and A71V in 2BPX (46M and 50L). For the phenotypic assay dataset the following background mutations were introduced: K7Q, I13V, G16E, K20I, I33F, M36L, S37N, I62V, I63H, A67C, A71V, G73S, I84V, L90M, and 95C in 3PWR for the genotype FB15; K7Q, I13V, G16E, K20I, I33F, M36L, K41R, I62V, P63H, V64I, A71V, G73S, I84V, and L90M in 3EL1 for the genotype FB15; K7Q, L10V, I13V, G16E, K20R, I33L, E35D, M36I, S37N, M46I, I54V, Q58E, I62V, I63H, I64V, A67C, V82F, I84V, and A95C in 3PWR for the genotype GH9; L10I, I13V, K20M, E35D, M36I, S37N, R41K, M46I, I54V, Q58E, H69K, V82M, and L89I in 1MUI for the genotype RU1; K7Q, L10V, I13V, R14K, K20M, E35D, M36I, M46I, I54V, Q58E, P63L, V64I, H69K, V82M, and L89I in 3EL1 for the genotype RU1; L10I, I13V, K20M, E35D, M36I, S37N, R41K, M46I, I54V, Q58E, H69K, V82M, and L89I in 1MUI for the genotype RU1; K7Q, L10I, I13V, R14K, L24I, L33F, K46L, I62V, A71V, T82A, V84I, and Q92K in 1K6C for the genotype iZ2; L10I, I13V, L24I, L33F, S37N, M46L, I62V, L63P, A71V, and V82A in 1MUI for the genotype iZ2; K7Q, L10I, I13V, R14K, L24I, L33F, K41R, M46L, I62V, V64I, A71V, V82A, and Q92K in 3EL1 for the genotype iZ2.

In the following, preparation for simulations of all the structures mentioned above is described in both holo and apo states, with the exception of structure 1HXB, 1MUI, and 2BPX for the HIVdb dataset, for which simulations in apo state were not performed (see "Equilibrium MD simulations and free energy calculations" section).

Remaining set up of the system in this study has been performed in the manner as described previously [17]. In short, the Gromacs simulation software package was used to set up (version 4.6.5), carry out, and analyse the MD simulations (versions 5.0.2 and 5.1.2) [68, 69]. The $pK_a$ of residues was predicted using Propka [70] and protease was assigned monoprotonated state on either D25/D25$^\prime$, here the prime refers to the second subunit of the protein. The Amber99SB*-ILDN force field was used for parametrisation of the protease. The Chemaxon Calculator [71] was used to determine inhibitor protonation, while Gaussian09 [72] was used to optimise inhibitor geometry and calculate electrostatic potential. Partial charges were assigned by performing restrained electrostatic potential fit [73]. The complex was solvated in TIP3P water molecules with 1.4 nm buffer in each dimension with 0.15 mol/l concentration of $\hbox {Cl}^-$ and $\hbox {Na}^+$ ions [74] to neutralise the system.

#### Equilibrium MD simulations and free energy calculations

The equilibrium MD simulations and the free energy calculations in this study have been performed in the manner as described previously [17]. In short, each system was subjected to steepest descent energy minimisation. Before equilibrium simulation, in order to avoid too close contacts between atoms, simulated annealing in length of 1 ns was performed for the following complexes: 1MUI (76V for the genotype iZ2) in D25$^\prime$ protonation state, 1MUI (76L for the genotype iZ2) in D25 protonation state, 2BPX (46V and 84I variants) in D25 protonation state, 3EKV (88S variant) in D25 protonation state, 3EL1 (76L and 76V for the genotype iZ2) in D25$^\prime$ protonation state. Ten replicas of 200 ns simulation for each complex were performed at 300 K. For all of the analyses that followed, the first 20 ns of the simulations were considered to be a part of the system equilibration process and thus discarded, with the exception of free energy calculations, where first 10 ns were discarded. The protocol for free energy calculations was adjusted from the non-equilibrium simulation approach used in assessing changes in protein thermal stabilities and protein–protein interactions upon amino acid mutation [75]. For calculating the free energy change upon mutation of apo structures, $\Delta G_1$, for inclusion in $\Delta \Delta G$ estimates for different inhibitors, in case of mutations M46I and I50L, wildtype apo structure 1HPV was used for the simulations as the background sequences for these mutations were the same. Similarly, for wildtype I84V as well as mutant M46I, I50L, and I84V simulations corresponding apo structure 1HPV variants were used for $\Delta \Delta G$ estimates for each mutation.

From each of the equilibrium simulations described above, trajectory frames were extracted equidistantly in time every 10 ns. After generating hybrid structures for every snapshot using the pmx [76] framework, short 20 ps simulations were performed to equilibrate velocities, after which alchemical transitions were carried out in 50 ps. Identical parameters were used for equilibrium simulations, equilibration, and alchemical transitions with soft-core potential for non-bonded interactions [77]. The Crooks Fluctuation Theorem [78] was used to relate the obtained work distributions to the free energy values by employing maximum likelihood estimator [79], with the error estimates obtained by the bootstrap approach. Simulations in both active site protonation states contributed to the free energy estimates, while for the rest of analysis reported in this study only the lowest free energy protonation state was used.

#### Partial least-squares regression

Partial least-squares regression was performed with the functional mode analysis tool [80] using the heavy atoms of protein as predictors. Wildtype and mutant protein simulation trajectories were labelled using constants 0 and 1 as target values, respectively.

For each mutation and inhibitor combination cross-validation (CV) was performed to verify the models. During CV, all trajectories for wildtype and mutant complexes were concatenated, superimposed to minimise the variance over the ensemble [81], and divided into five equal parts. For every iteration, a model was trained on four parts of labelled input in equal proportions from wildtype and mutant simulations, after which it was used to make prediction for the last part. The Pearson correlation between the actual and predicted labels was used to evaluate the quality of the model. Number of components $i = 1, \dots , 25$ was tested in each iteration. For the final model, the number of components was chosen from the correlation curve in CV such that choosing a higher number of components only marginally improves the performance of the model.

#### Estimation of resistance factor change from free energy of inhibitor binding change

Cheng-Prusoff equation [29] relates inhibitor’s $IC_{50}$ and binding affinity $K_i$, which in turn can be estimated from inhibitor binding free energy $\Delta G$:

1 $$\begin{aligned} IC_{50}= K_i\bigg (1+\frac{[S]}{K_m}\bigg ) = e^\frac{\Delta G}{k_B T}\bigg (1+\frac{[S]}{K_m}\bigg )\,, \end{aligned}$$

where [S] is a fixed substrate concentration, $K_m$ is the concentration of the substrate at which the enzyme is at its half-maximal activity, $k_B$ is the Boltzmann constant, and T is the absolute temperature. Thus, given two RF values for two proteases with sequences *A* and *B*, $RF_A$ and $RF_B$, their ratio can be related to $\Delta \Delta G$:

2 $$\begin{aligned} RF_R = \frac{RF_A}{RF_B} = \frac{e^\frac{\Delta G_A}{k_BT}}{e^\frac{\Delta G_B}{k_BT}}\Bigg (\frac{1+\frac{[S]}{K_m^A}}{1+\frac{[S]}{K_m^B}}\Bigg ) = e^{\frac{\Delta \Delta G}{k_BT}}\Bigg (\frac{1+\frac{[S]}{K_m^A}}{1+\frac{[S]}{K_m^B}}\Bigg ) = e^{\frac{\Delta \Delta G}{k_BT}} C\,. \end{aligned}$$

We are interested in obtaining a distribution of the $RF_R$ values after calculating the double free energy differences $\Delta \Delta G$:

3 $$\begin{aligned} p(RF_R |\Delta \Delta G, C) \propto p(\Delta \Delta G, C|RF_R)p(RF_R) \end{aligned}$$

When there are multiple $RF_R$ measurements and $\Delta \Delta G$ calculations available for the wildtype and mutant protein complexed with different ligands, *C* can be expressed as a function of the available values $C = C_i(\Delta \Delta G_i , RF_R^i), i = 1, \dots , n$. This gives the final posterior distribution:

4 $$\begin{aligned} p(RF_R |\Delta \Delta G, \Delta \Delta G_i , RF_R^i) \propto p(\Delta \Delta G, \Delta \Delta G_i , RF_R^i | RF_R) p(RF_R), \end{aligned}$$

where $C_i = RF_R^i e^\frac{-\Delta \Delta G_i}{k_B T}$. The $\Delta \Delta G$ values are sampled from a Gaussian distribution with the mean and standard deviation corresponding to the calculated double free energy difference and estimated error, respectively.

### Phenotypic assay for resistance factor value estimation

The experimental data on L76V resistance is based on samples of patients who underwent multiple therapy failures with different PIs. Those variants were observed in the diagnostic procedure, sequenced, and subsequently tested in a phenotypic assay as described by Walter et al. [82]. The tests were carried out after the patient’s variant was cloned into a recombinant derivate of the HIV NL4-3, called pNL4-3-Delta-PRT5. The L76V mutation was reverted to wildtype by site directed mutagenesis. This allowed to determine the effect of the genetic background upon the L76V. These variants were analysed in cell culture experiments where they were exposed to different PIs in different concentrations to estimate their RF values (Table 3). Based on these variants, the clones were specifically modified by site-directed mutagenesis so that different variants of L76V could be tested in different genetic backgrounds. For simplicity, regardless of the residue at position 76 of protease as present in the original clinical samples, in the context of this paper L76 is referred to as wildtype residue and V76 as the mutant residue as per reference sequence HXB2.

## Supplementary information

**Additional file 1: Table S1.** The names of the isolates, whose RF data was used in this study, as reported in HIVdb, and the reference of the study where RF measurements were performed.** Table S2.** Inhibitor binding free energy change upon switching the proton from the reference protonated active site residue to the active site residue on the opposite subunit for wildtype and mutant proteins. ± shows bootstrap error estimate, all values in kcal/mol.** Table S3.** Average hydrogen bonds number between residues D30, T31, and T74 with N88 and S88 for wildtype and mutant complexes, respectively. Columns 3 and 4 of the table corresponds to hydrogen bonds within monomer A of protease and columns 5 and 6 of the table corresponds to hydrogen bonds within monomer B of protease (residues marked with prime symbol). ± indicates standard error of bond frequency across independent simulations.** Table S3.** Inhibitor binding free energy change upon switching the proton from the reference protonated active site residue to the active site residue on the opposite subunit for wildtype and mutant proteins. ± shows bootstrap error estimate, all values in kcal/mol.** Figure S3.** Convergence of the* RF*_*R*_ estimates. The shaded areas show the 95% credible interval.** Figure S4.** Interpolation between the extremes of the FMA models for the corresponding complexes. Blue-to-magenta bands correspond to the interpolation along the mode as represented as cartoon for backbone and as sticks for residues 30, 45, and 58, with blue corresponding to L76 state and magenta to V76 state. Mutated residue 76 is not part of the model and is represented here as gray dash.** Table S4.** Inhibitor binding free energy change upon switching the proton from the reference protonated active site residue to the active site residue on the opposite subunit for wildtype and mutant proteins. ± shows bootstrap error estimate, all values in kcal/mol.** Figure S5.** Energy differences of non-bonded interactions between protein and inhibitor in wildtype and mutant complexes. Only residues, for which the difference between the wildtype and the mutant complexes is higher than the propagated error and its absolute value higher than 0.1 kcal/mol are shown.

## Data Availability

The datasets used and/or analysed during the current study are available from the corresponding author on reasonable request.

## Abbreviations

AIDS: Acquired immune deficiency syndrome
APV: Amprenavir
ART: Antiretroviral therapy
ATV: Atazanavir
CV: Cross-validation
DRV: Darunavir
FMA: Functional mode analysis
FPV: Fosamprenavir
HIV-1: Human immunodeficiency virus type 1
IDV: Indinavir
LPV: Lopinavir
MD: Molecular dynamics
NFV: Nelfinavir
PDB: Protein Data Bank
PI: Protease inhibitor
PLS: Partial least-squares
RAM: Resistance-associated mutation
RF: Resistance factor
SQV: Saquinavir
TPV: Tipranavir
