# Supplementary material for: Non-active site mutants of HIV-1 protease influence resistance and sensitisation towards protease inhibitors
Source: Retrovirology. 2020 May 19;17:13. doi: 10.1186/s12977-020-00520-6 (PMC7236880; doi:10.1186/s12977-020-00520-6)
Supplement: Supplementary file 1 — Additional file 1: Table S1. The names of the isolates, whose RF data was used in this study, as reported in HIVdb, and the reference of the study where RF measurements were performed. Table S2. Inhibitor binding free energy change upon switching the proton from the reference protonated active site residue to the active site residue on the opposite subunit for wildtype and mutant proteins. ± shows bootstrap error estimate, all values in kcal/mol. Table S3. Average hydrogen bonds number between residues D30, T31, and T74 with N88 and S88 for wildtype and mutant complexes, respectively. Columns 3 and 4 of the table corresponds to hydrogen bonds within monomer A of protease and columns 5 and 6 of the table corresponds to hydrogen bonds within monomer B of protease (residues marked with prime symbol). ± indicates standard error of bond frequency across independent simulations. Table S3. Inhibitor binding free energy change upon switching the proton from the reference protonated active site residue to the active site residue on the opposite subunit for wildtype and mutant proteins. ± shows bootstrap error estimate, all values in kcal/mol. Figure S3. Convergence of the RFR estimates. The shaded areas show the 95% credible interval. Figure S4. Interpolation between the extremes of the FMA models for the corresponding complexes. Blue-to-magenta bands correspond to the interpolation along the mode as represented as cartoon for backbone and as sticks for residues 30, 45, and 58, with blue corresponding to L76 state and magenta to V76 state. Mutated residue 76 is not part of the model and is represented here as gray dash. Table S4. Inhibitor binding free energy change upon switching the proton from the reference protonated active site residue to the active site residue on the opposite subunit for wildtype and mutant proteins. ± shows bootstrap error estimate, all values in kcal/mol. Figure S5. Energy differences of non-bonded interactions between protein and inhibitor in wildt [file 12977_2020_520_MOESM1_ESM.pdf]

Supplementary material

Table S1: The names of the isolates, whose RF data was used in this study, as reported in HIVdb, and the reference of the study where RF measurements were performed.

**Mutation**		**Isolate**	**Reference**
**M46I**	wildtype	71V-11, A71V-7, Bru-A71V-3	[1]
	mutant	P372	[2]
**I50L**	wildtype	71V-11, A71V-7, Bru-A71V-3	[1]
	mutant	71V-12, A71V-8, BruA71V-4	[1]
**I84V**	wildtype	JGP-M1C	[3]
	mutant	JGP-M2C, JGP-M2R	[3]
**N88S**	wildtype	RZ27 (IDV), RZ28 (FPV)	[4]
	mutant	RZ22 (IDV), RZ-L4 (FPV)	[4]

Table S2: Inhibitor binding free energy change upon switching the proton from the reference protonated active site residue to the active site residue on the opposite subunit for wildtype and mutant proteins. ± shows bootstrap error estimate, all values in kcal/mol.

Inhibitor	Genotype	Reference protonated state	$\Delta\Delta G^{prot}_{WT}$	$\Delta\Delta G^{prot}_{MUT}$
APV	M46I	D25′	−1.86 ± 0.23	−2.32 ± 0.21
IDV	M46I	D25	1.15 ± 0.27	0.53 ± 0.45
APV	I50L	D25′	−1.74 ± 0.31	−1.85 ± 0.18
IDV	I50L	D25	1.64 ± 0.23	0.52 ± 0.38
APV	I84V	D25′	−1.67 ± 0.25	−0.31 ± 0.27
IDV	I84V	D25	1.67 ± 0.44	1.29 ± 0.38
LPV	I84V	D25′	−0.6 ± 0.35	−1.33 ± 0.3
SQV	I84V	D25	1.03 ± 0.19	2.04 ± 0.27
APV	N88S	D25	2.23 ± 0.29	−0.16 ± 0.24
IDV	N88S	D25	2.22 ± 0.51	1.42 ± 0.45

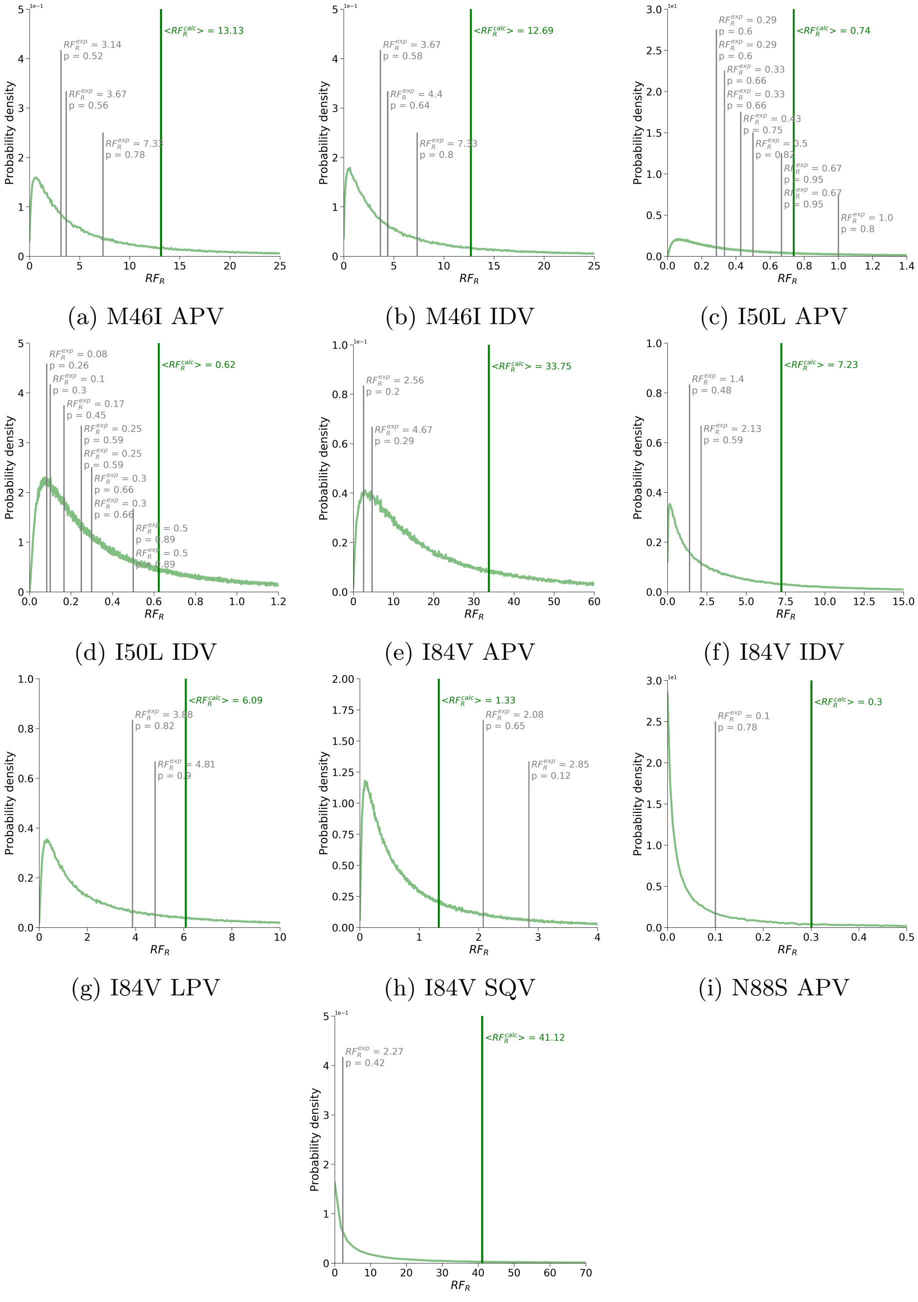

(a) M46I APV (b) M46I IDV (c) I50L APV

(d) I50L IDV (e) I84V APV (f) I84V IDV

(g) I84V LPV (h) I84V SQV (i) N88S APV

(j) N88S IDV

Figure S1: Calculated RF_R distributions and experimental estimates. p designates the proportion of RF_R^{calc} at least as extreme as RF_R^{exp} compared to mean RF_R^{calc}. *Nota bene:* in case of APV, RF_R^{exp} measurements are for its prodrug FPV.

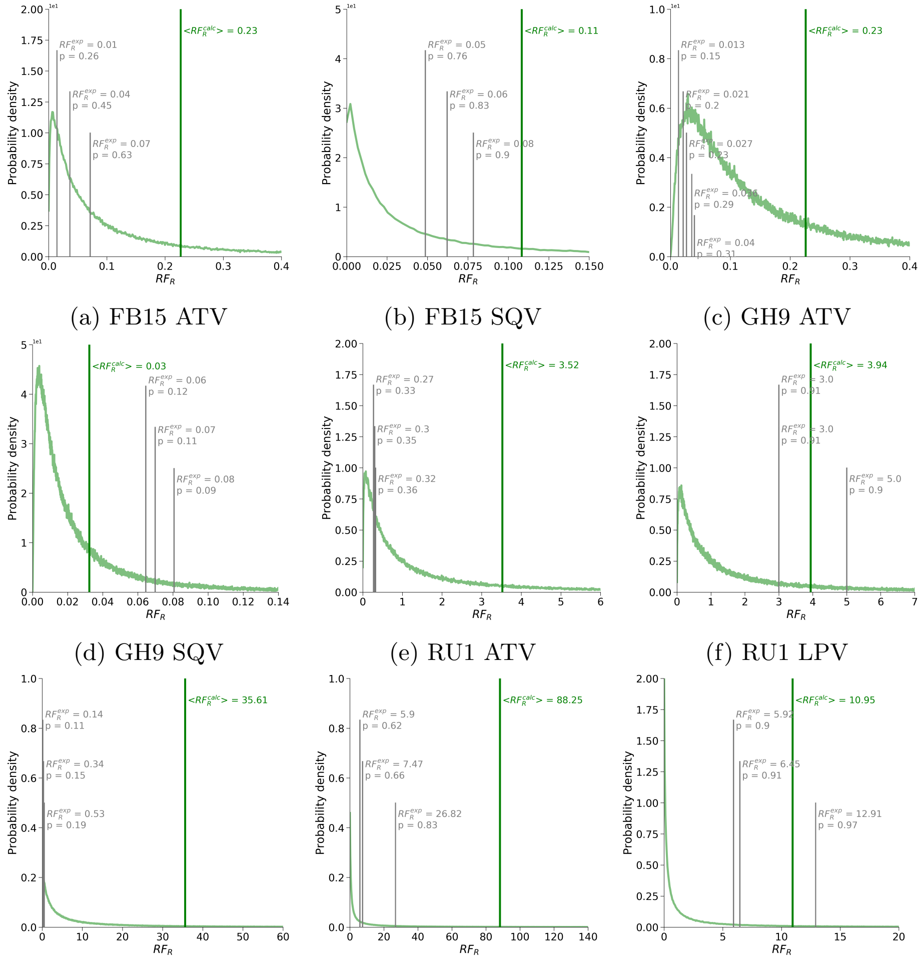

(a) FB15 ATV (b) FB15 SQV (c) GH9 ATV

(d) GH9 SQV (e) RU1 ATV (f) RU1 LPV

(g) iZ2 ATV (h) iZ2 IDV (i) iZ2 LPV

Figure S2: Calculated RF_R distributions and experimental estimates. p designates the proportion of RF_R^{calc} at least as extreme as RF_R^{exp} compared to mean RF_R^{calc}. *Nota bene:* in case of APV, RF_R^{exp} measurements are for its prodrug FPV.

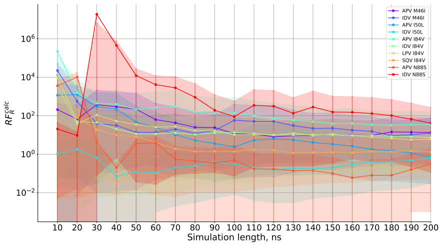

(a) M46I, I50L, I84V, N88S

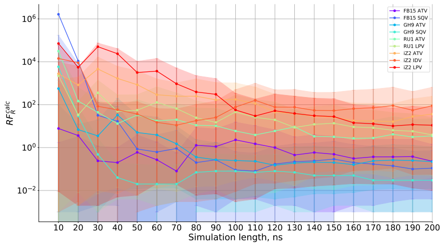

(b) L76V

Figure S3: Convergence of the RF_R estimates. The shaded areas show the 95% credible interval.

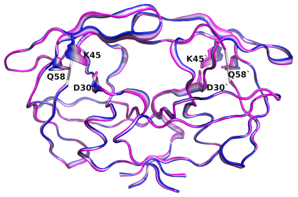

(a) FB15 ATV

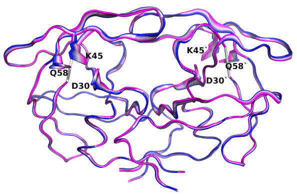

(b) FB15 SQV

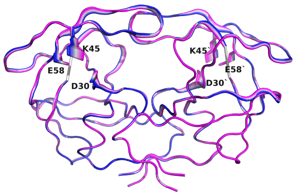

(c) GH9 ATV

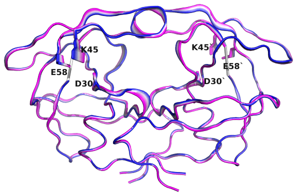

(d) GH9 SQV

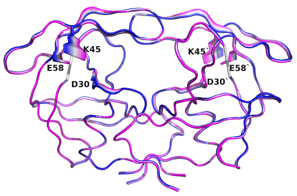

(e) RU1 ATV

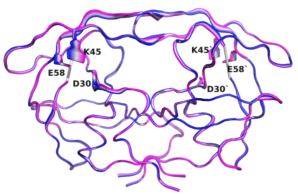

(f) RU1 LPV

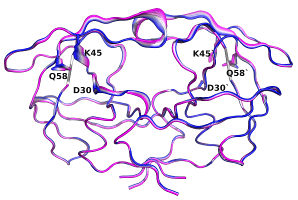

(g) iZ2 ATV

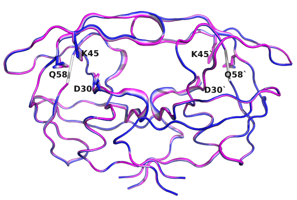

(h) iZ2 IDV

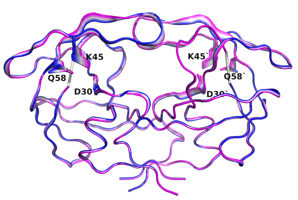

(i) iZ2 LPV

Figure S4: Interpolation between the extremes of the FMA models for the corresponding complexes. Blue-to-magenta bands correspond to the interpolation along the mode as represented as cartoon for backbone and as sticks for residues 30, 45, and 58, with blue corresponding to L76 state and magenta to V76 state. Mutated residue 76 is not part of the model and is represented here as gray dash.

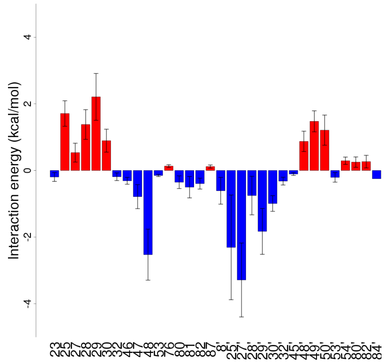

(a) FB15 ATV

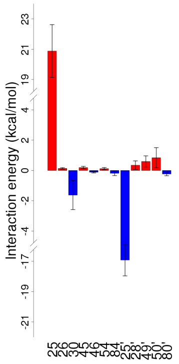

(b) FB15 SQV

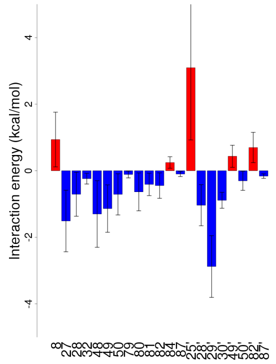

(c) GH9 ATV

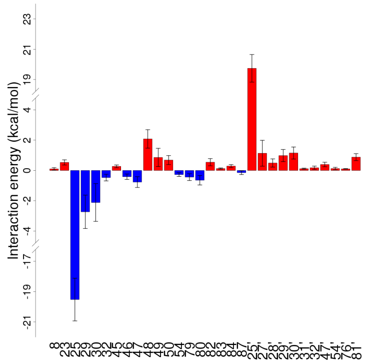

(d) GH9 SQV

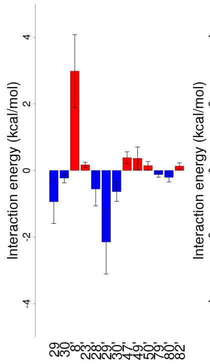

(e) RU1 ATV

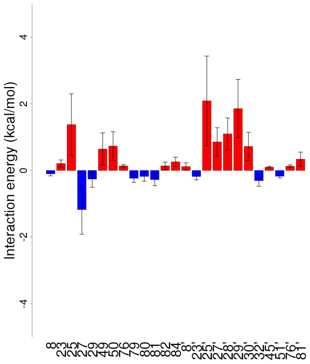

(f) RU1 LPV

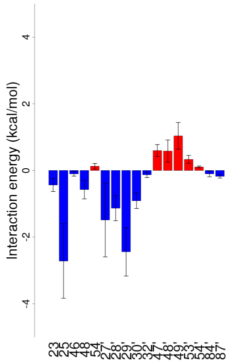

(g) iZ2 ATV

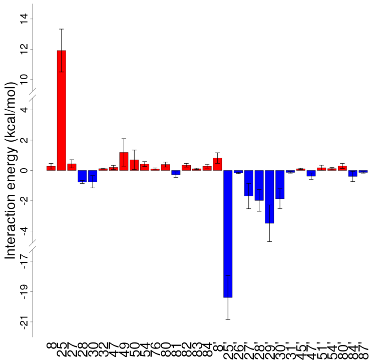

(h) iZ2 IDV

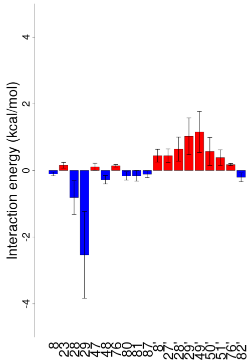

(i) iZ2 LPV

Figure S5: Energy differences of non-bonded interactions between protein and inhibitor in wildtype and mutant complexes. Only residues, for which the difference between the wildtype and the mutant complexes is higher than the propagated error and its absolute value higher than 0.1 kcal/mol are shown.

Table S3: Average hydrogen bonds number between residues D30, T31, and T74 with N88 and S88 for wildtype and mutant complexes, respectively. Columns 3 and 4 of the table corresponds to hydrogen bonds within monomer A of protease and columns 5 and 6 of the table corresponds to hydrogen bonds within monomer B of protease (residues marked with prime symbol). ± indicates standard error of bond frequency across independent simulations.

**Residues**	**Inhibitor**	**N88**	**S88**	**N88′**	**S88′**
**D30/D30′**	**APV**	0.005 ± 2×10^{-4}	0.61 ± 0.05	5×10^{-4} ± 3×10^{-7}	0.66 ± 0.12
	**IDV**	2×10^{-4} ± 2×10^{-7}	0.22 ± 0.008	0.001 ± 2×10^{-6}	0.55 ± 0.08
**T31/T31′**	**APV**	1.28 ± 0.004	0.21 ± 0.03	1.56 ± 0.003	0.13 ± 0.04
	**IDV**	1.26 ± 0.007	0.47 ± 0.03	1.35 ± 0.008	0.27 ± 0.04
**T74/T74′**	**APV**	0.8 ± 0.003	3×10^{-4} ± 2×10^{-7}	0.85 ± 0.001	2×10^{-4} ± 2×10^{-7}
	**IDV**	0.71 ± 0.001	2×10^{-4} ± 2×10^{-7}	0.79 ± 0.001	3×10^{-4} ± 9×10^{-7}

Table S4: Inhibitor binding free energy change upon switching the proton from the reference protonated active site residue to the active site residue on the opposite subunit for wildtype and mutant proteins. ± shows bootstrap error estimate, all values in kcal/mol.

Inhibitor	Genotype	Reference protonated state	$\Delta\Delta G_{WT}^{prot}$	$\Delta\Delta G_{MUT}^{prot}$
ATV	FB15	D25′	−1.17 ± 0.31	−2.08 ± 0.36
SQV	FB15	D25	−0.08 ± 0.25	0.4 ± 0.37
ATV	GH9	D25′	−0.13 ± 0.21	−1.23 ± 0.32
SQV	GH9	D25	0.45 ± 0.21	−0.08 ± 0.31
ATV	RU1	D25′	−4.21 ± 0.38	−1.76 ± 0.47
LPV	RU1	D25′	−1.41 ± 0.32	−0.75 ± 0.37
ATV	iZ2	D25	−0.82 ± 0.34	−1.25 ± 0.4
IDV	iZ2	D25′	−0.95 ± 0.44	1.43 ± 0.31
LPV	iZ2	D25	0.56 ± 0.27	0.5 ± 0.75

[1] R. Colonno, R. Rose, C. McLaren, A. Thiry, N. Parkin, J. Friborg, Identification of I50L as the signature atazanavir (ATV)-resistance mutation in treatment-naive HIV-1-infected patients receiving ATV-containing regimens, J. Infect. Dis. 189 (10) (2004) 1802–1810. doi:10.1086/386291.

[2] C. J. Petropoulos, N. T. Parkin, K. L. Limoli, Y. S. Lie, T. Wrin, W. Huang, H. Tian, D. Smith, G. A. Winslow, D. J. Capon, J. M. Whitcomb, A novel phenotypic drug susceptibility assay for human immunodeficiency virus type 1, Antimicrob. Agents Chemother. 44 (4) (2000) 920–928. doi:10.1128/aac.44.4.920-928.2000.

[3] J. G. Prado, T. Wrin, J. Beauchaine, L. Ruiz, C. J. Petropoulos, S. D. Frost, B. Clotet, T. D. Richard, J. Martinez-Picado, Amprenavir-resistant HIV-1 exhibits lopinavir cross-resistance and reduced replication capacity, AIDS 16 (7) (2002) 1009–1017. doi:10.1097/00002030-200205030-00007.

[4] R. Ziermann, K. Limoli, K. Das, E. Arnold, C. J. Petropoulos, N. T. Parkin, A mutation in human immunodeficiency virus type 1 protease, N88S, that

causes in vitro hypersensitivity to amprenavir, J. Virol. 74 (9) (2000) 4414–4419. doi:10.1128/JVI.74.9.4414-4419.2000.
